# You are not alone…Polish Psychiatric Association supporting Ukraine

**DOI:** 10.1192/j.eurpsy.2023.180

**Published:** 2023-07-19

**Authors:** J. Samochowiec

**Affiliations:** Department of Psychiatry, Pomeranian Medical University in Szczecin, Szczecin, Poland

## Abstract

**Abstract:**

Immediately after Russia commenced aggression against Ukraine, the Polish Psychiatric Association publicly called for humanitarian support for Ukraine and initiated meetings with representatives of Psychiatric Societies operating in Ukraine in order to identify current needs in the war-stricken areas and coordinate aid.

The PPA allocated its financial resources to humanitarian aid and, through the EPA, appealed for condemnation of military operations as well as support for Ukraine by individual NPAs.

According to the UHNR data over 4 million displaced people, refugees, came to Poland so far and some of them benefited from such help.

The Polish Psychiatric Association supports the initiatives of non-governmental organizations supporting refugees and monitors and responds to the needs reported by the Ukrainian side on an ongoing basis. At present, the PPA activities focus on the following priorities:

1. Need-adapted-help: Provision of customized aid - not only medications but also power generators, technical equipment. And so, the PPA shipped to Ukraine some basic equipment, sleeping mats, bedding, mattresses, backpacks, cleaning products, personal hygiene products, as well as tools for renovation and construction.

2. Awareness - highlighting the consequences of Russia’s aggression on people with mental disorders in Ukraine and its impact on the entire population (PTSD, grief ). Inviting and lobbying for dissemination of personal reports of Ukrainian health care workers and patients at international conventions, forums, events

3. Empowerment of personnel - strengthening competences required in provision of assistance in war-related disorders, training, projects of activities both across Poland and Ukraine

4. Supporting and responding to the needs reported by local psychiatric assistance centers facilitating and strengthening the competence of personnel in helping refugees

**Disclosure of Interest:**

None Declared

